# Prevalence of HSV‐1/HSV‐2 and CMV Infections in Infertile Men and Their Impact on Sperm Parameters: A Cross‐Sectional Study in Iran

**DOI:** 10.1002/hsr2.70221

**Published:** 2025-02-11

**Authors:** Seyed Mansour Salar, Fahime Edalat, Arash Letafati, Seyed Mehdi Kalantar, Fatemeh Javanmardi, Ahmad najafi, Mahmoud vakili, Afagh Moattari

**Affiliations:** ^1^ Department of Bacteriology & Virology, School of Medicine Shiraz University of Medical Sciences Shiraz Iran; ^2^ Autophagy Research Center Shiraz University of Medical Science Shiraz Iran; ^3^ Department of Virology, Faculty of Public Health Tehran University of Medical Sciences Tehran Iran; ^4^ Research Center for Clinical Virology Tehran University of Medical Science Tehran Iran; ^5^ Reproductive & Genetic Unit, Recurrent Abortion Research Center, Yazd Reproductive Science Institute Yazd University of Medical Sciences Yazd Iran; ^6^ Department of Biostatistics Shiraz University of Medical Sciences Shiraz Iran; ^7^ Department of Immunology Shahid Sadoughi University of Medical Sciences Yazd Iran; ^8^ Health Monitoring Research Center, School of Medicine Shahid Sadoughi University of Medical Sciences Yazd Iran

**Keywords:** cytomegalovirus, herpes simplex, male infertility, seminal fluid, viral infection

## Abstract

**Background and Aims:**

Viral infections are significant contributors to infertility. This study aimed to determine the prevalence of Herpes Simplex Virus 1 and 2 (HSV‐1/HSV‐2) and cytomegalovirus (CMV) infections in men with infertility, compared to a control group of fertile men.

**Methods:**

A cross‐sectional study was conducted with seminal fluid samples from 100 fertile and 100 infertile men, aged 20–40 years, referred to Yazd Infertility Center. DNA extraction was done using the AmpliSens kit, and the presence of CMV and HSV‐1/HSV‐2 was determined via real‐time PCR. Sperm parameters (count, quick & slow, motility, morphology, and volume) were also assessed and compared to the control group.

**Results:**

CMV infection was found in 16% of fertile and 10% of infertile men. HSV‐1/HSV‐2 prevalence was significantly higher in infertile men (32%) compared to fertile men (8%). Abnormal sperm count was significantly associated with HSV status (*p* = 0.001), as was abnormal morphology (*p* = 0.021), while abnormal quick & slow, motility and volume showed no significant association. Data analysis indicated no correlation between sperm parameters—including sperm quick & slow, motility, count, morphology, and semen volume—and CMV infection.

**Conclusion:**

HSV infection is linked to sperm‐specific parameters, particularly count and morphology. These findings enhance our understanding of viral‐induced male infertility and underscore the need for targeted approaches to manage HSV‐1/HSV‐2‐related infertility.

## 1 Introduction

Infertility stands as a significant medical challenge, particularly concerning male reproductive health. In the context of men, infertility is defined as the incapacity to facilitate pregnancy in a fertile female partner, persisting for a year of regular sexual activity without resulting in conception. This prolonged lack of pregnancy following the aforementioned timeframe underscores the intricate complexities that can hinder successful reproduction [1]. Male infertility encompasses a spectrum of contributory elements, notably including structural impairment within the male reproductive system, the presence of varicocele, obstruction within the genital tract, as well as the influence of endocrine and metabolic disorders. Particularly noteworthy are bacterial and viral infections, which can profoundly impact seminal fluid, testicular health, venous structures, and reproductive glands. These multifaceted factors collectively underscore the intricate nature of male infertility and its multifarious origins [2]. Infections, operating through diverse mechanisms, have the potential to disrupt male infertility dynamics. This disruption encompasses interference with the spermatogenesis process, impairment of sperm function, and the potential to induce obstructions within the genital tract [3, 4].

Different types of microorganisms, such as bacteria, viruses, and protozoa, have the potential to invade the male reproductive system and hinder male fertility. Additionally, a significant number of these harmful agents can be transmitted through sexual contact [5]. The presence of microbial pathogens can lead to detrimental consequences by directly interacting with sperm cells. These consequences encompass actions like causing sperm cell demise, diminishing sperm count, and impeding their movement. Furthermore, the activation of inflammatory cytokines due to microbial infections might indirectly disturb sperm production and disrupt the proper function of reproductive organs, ultimately resulting in a negative impact on male fertility [6, 7]. *Herpesviridae* is considered a significant contributor to the risk of infertility [8]. Proposed findings indicate that infections caused by this family, especially herpes simplex virus type 1 and 2 (HSV‐1, HSV‐2) and Cytomegalovirus (CMV) alongside other viruses such as Human Papillomavirus (HPV), Hepatitis B Virus (HBV), Hepatitis C Virus (HCV), Human Immunodeficiency Virus (HIV), and Adeno‐Associated Virus (AAV) have detrimental impacts on the male reproductive system. Employing sperm‐washing techniques can effectively prevent the transmission of these viruses [9, 10, 11].

The aim of this study was to investigate the prevalence of HSV‐1/HSV‐2 and CMV infections in infertile and fertile men. The investigation also embarked on a comprehensive exploration of sperm quality, meticulously scrutinizing factors such as sperm count, motility, morphology, and volume.

## 2 Materials and Methods

A total of 200 clinical specimens containing 100 samples of seminal fluid from infertile men and 100 samples of seminal fluid from healthy men who were similar in age were collected from the Infertility Research Center of Shahid Sadoughi University of Medical Sciences in Yazd‐Iran (Ethical code: IR.SSU.RSI.REC.1395.32). For each participant, a consent letter was provided and if they had any other concurrent infections (related to viruses or bacterial infections) were excluded from the study. After providing the consent form and giving the necessary explanations to the subjects, a seminal fluid sample was taken from each person for the analysis of sperm as well as molecular analysis of sperm. The criteria used to distinguish the fertile and infertile groups were according to the World Health Organization (WHO) criteria [12]. Seminal fluid samples were mixed with an equal volume of PBS buffer and transferred to the laboratory at a temperature of 37°C in cryotubes free from RNase and Dnase.

Following an assessment of the visual characteristics and consistency of seminal fluid, along with the quantification of its volume, a 10 μL sample was carefully placed on neobar lam for subsequent sperm analysis. This analysis encompassed quantifying sperm count per milliliter and evaluating sperm motility categories: progressive (rapid and slow), stationary, and nonmotile. Subsequently, a suspension containing seminal fluid was meticulously prepared, and following fixation and staining, it was utilized for a comprehensive examination of sperm morphology. Extraction of the seminal fluid DNA was carried out by AmpliSens kit (Russia), and then the DNA that was extracted was kept until the test was carried out in −80°C. Real‐time PCR was used to detect genomic sequences of HSV‐1/HSV‐2 and CMV viruses (AmpliSens, Russia) according to Kit instructions. The concentration of the duplicated product was measured using the PCR process and the labeled probes. The presence of CMV was indicated by the increase in fluorescence of the fluorophores FAM and JOE and the HSV‐1/HSV‐2 virus was shown by fluorescence of the fluorophores FAM/GREEN and JOE/YELLOW/HEX.

The detection kit uses the Hot Start method to minimize nonspecific responses and assures maximum sensitivity. Fifteen microliters of the kit of real‐time PCR master mix and 10 μL of extracted DNA were mixed, which eventually reached a final volume of 25 μL (Table 1). It is worth noting that because of the sensitivity of fluorescence materials to light, all of the steps of mixing these materials were performed in a relatively dark environment.

**Table 1:** Temperature program for real‐time PCR.

	Plate‐type instruments		
Step	Temperature	Time	Cycle
1	95°C	15 min	1 Round
2	95°C	5 s	
	60°C	20 s	5 Round
	72°C	15 s	
3	95°C	5 s	
	60°C	30 s	
		Fluorescence detection	40 Round
	72°C	15 s	

## 3 Statistical Analysis

Descriptive statistics were computed for all variables, with means ± standard deviations reported for continuous variables, and frequencies and percentages for categorical variables. The Shapiro–Wilk test assessed continuous variable normality. Independent samples *t*‐tests were used to compare group mean differences. Chi‐square tests assessed variable associations for categorical data. Cohen's d widely used to measure the effect size that quantifies the magnitude of difference between two groups. A commonly used interpretation is to refer to effect sizes as small (*d* < 0.2), medium (*d* = 0.5), and large (*d* > 0.8).

By adhering to these analytical methodologies, the research aimed to unravel comprehensive insights into seminal fluid attributes, sperm motility, and morphology, thus advancing our understanding of reproductive health. In this research, a *p*‐value lower than 0.05 was considered statistically significant. The Statistical Package for the Social Sciences version 22 (SPSS 22) is used for statistical analysis.

## 4 Results

**Figure 1:**
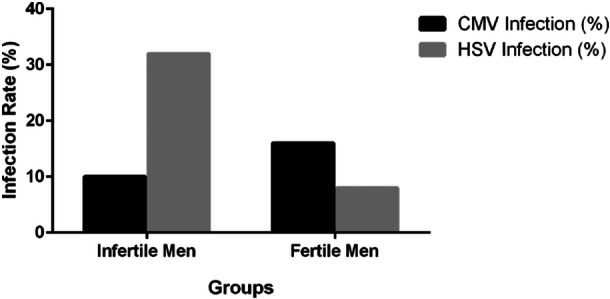
Prevalence of HSV‐1/HSV‐2 and CMV infections in infertile and fertile individuals. The analysis indicated no significant difference in CMV infection rates between fertile and infertile men (*p*‐value = 0.37). However, there was a statistically significant difference in the prevalence of HSV‐1/HSV‐2 infection between fertile and infertile men (*p*‐value = 0.003).

The research was conducted as a cross‐sectional study, involving a sample size of 100 individuals per group. Participants, aged between 20 and 40 years, were carefully selected with an age‐matching strategy to ensure both groups were comparable. The study assessed the prevalence of CMV infection among the participants, revealing that 10 out of the 100 infertile men (10%) tested positive for CMV. In the group of fertile men, 16 cases (16%) of infection with the same virus were identified. The results indicated no significant difference in CMV infection rates between fertile and infertile men (*p*‐value = 0.37). However, regarding HSV‐1/HSV‐2 infections, 32 out of 100 infertile men (32%) were infected, compared to 8 infected fertile men (8%). Statistical analysis showed a significant difference between fertile and infertile men in the prevalence of HSV1/HSV2 infection (*p*‐value = 0.003)(Figure 1).

**Figure 2:**
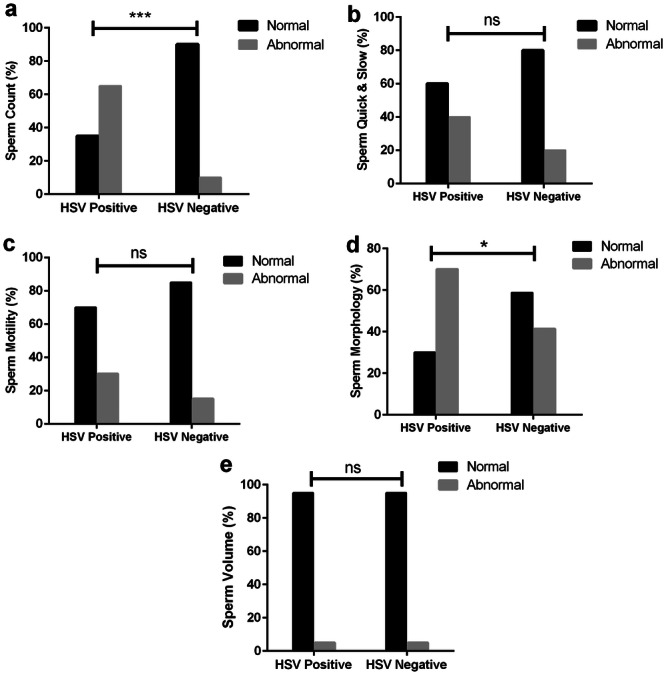
The study divided participants into two groups based on their HSV status: HSV negative and HSV positive. The analysis of sperm parameters yielded the following results: For the HSV‐negative group, abnormal sperm parameters were observed in 10% for count (a), 20% for quick & slow (b), 15% for motility (c), 41.3% for morphology (d), and 5% for volume (e). In contrast, normal sperm parameters were found in 90% for count (a), 80% for quick & slow (b), 85% for motility (c), 58.8% for morphology (d), and 95% for volume (e). In the HSV‐positive group, higher percentages of abnormal sperm parameters were noted: 65% for count (a), 40% for quick & slow (b), 30% for motility (c), 70% for morphology (d), and 5% for volume (e). Conversely, lower percentages of normal sperm parameters were observed, with 35% for count (a), 60% for quick & slow (b), 70% for motility (c), 30% for morphology (d), and 95% for volume (e). The *p*‐values indicated a high level of statistical significance, 0.001 for abnormal count (a) and 0.021 for abnormal morphology (d). No significant (ns) relationship was found for the other sperm parameters.

**Figure 3:**
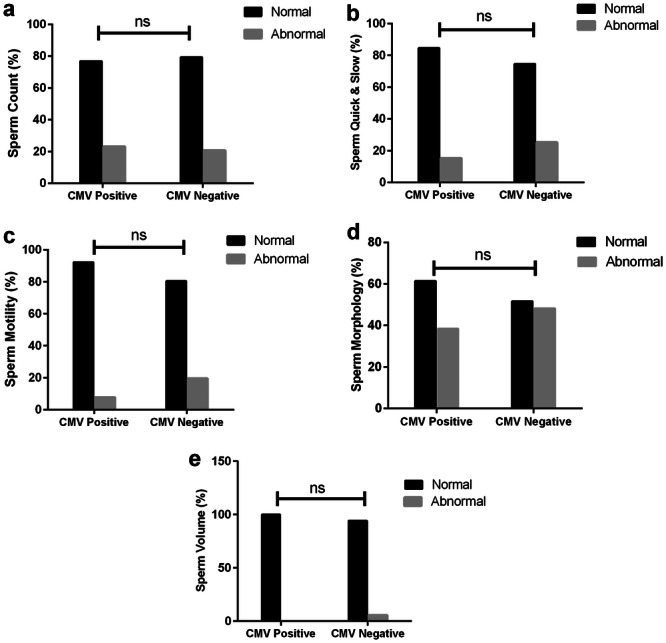
Percentage of normal and abnormal sperm parameters divided by CMV negative and positive individuals. CMV‐negative individuals had 36 (20.7%) abnormal and 138 (79.3%) normal sperm counts, while CMV‐positive individuals had 6 (23.1%) abnormal and 20 (76.9%) normal sperm counts (a). In the case of quick & slow, CMV‐negative individuals had 44 (25.3%) abnormal and 130 (74.7%) normal sperm quick & slow, whereas CMV‐positive individuals had 4 (15.4%) abnormal and 22 (84.6%) normal sperm quick & slow (b). In terms of motility, CMV‐negative individuals had 34 (19.5%) abnormal and 140 (80.5%) normal sperm motility, whereas CMV‐positive individuals had 2 (7.7%) abnormal and 24 (92.3%) normal sperm motility (c). Regarding morphology, CMV‐negative individuals exhibited 84 (48.3%) abnormal and 90 (51.7%) normal sperm morphology, while CMV‐positive individuals had 10 (38.5%) abnormal and 16 (61.5%) normal sperm morphology (d). In the case of volume, CMV‐negative individuals had 10 (5.7%) abnormal and 164 (94.3%) normal sperm volume, whereas CMV‐positive individuals had no abnormal volume (0%) and 26 (100%) normal sperm volume (e). The *p*‐values for these comparisons were not significant (ns).

The study examines the relationship between sperm parameters and CMV and HSV‐1/HSV‐2 infections. The results indicated that 65% of individuals who tested positive for HSV‐1/HSV‐2 had an abnormal sperm count, compared to only 10% of those who were HSV‐1/HSV‐2 negative (*p*‐value = 0.001). Additionally, 70% of HSV‐1/HSV‐2 positive patients exhibited abnormal sperm morphology, whereas 41.3% of HSV‐1/HSV‐2 negative individuals showed abnormal morphology (*p*‐value = 0.021) (Figure 2). Data analysis indicated no correlation between sperm parameters—including sperm quick & slow, motility, count, morphology, and semen volume—and CMV infection (Figure 3).

Also, sperm quality parameters and related variables in the infertile group were analyzed based on the presence or absence of CMV and HSV‐1/HSV‐2 infections. According to the *p*‐value and Cohen's d effect size, the count of sperm was significantly different between the HSV‐1/HSV‐2 negative and positive group (*p*‐value < 0.001, and effect size is more than 1 which shows a large effect size). No significant relationship was found for the other sperm parameters (Table 2). Moreover, no significant differences in sperm parameters were observed between CMV‐positive and CMV‐negative infertile individuals (Table 3).

**Table 2:** Quantitative sperm parameters in HSV‐1/HSV‐2‐positive and ‐negative infertile individuals.

Sperm features	HSV negative	HSV positive	*p*‐value (two‐tailed)	Effect size (Cohen's d)	*t*‐statistics
	*N* = 68	*N* = 32			
Count	52.09 ± 36.519^a^	19.81 ± 25.980	< 0.001	1.018	4.554
Quick & Slow	31.76 ± 18.064	32.44 ± 19.294	0.90	−0.036	−0.749
Motility	41.35 ± 19.175	40.38 ± 20.597	0.87	0.048	−0.325
Morphology	1.62 ± 0.853	1.75 ± 1.693	0.71	−0.097	0.872
Volume	3.385 ± 1.695	3.400 ± 1.111	0.97	−0.013	1.019

^a^ Data are represented as mean ± standard deviation.

**Table 3:** Quantitative sperm parameters in CMV‐positive and ‐negative infertile individuals.

Sperm features	CMV negative	CMV positive	*p*‐value (two‐tailed)	Effect size (Cohen's d)	*t*‐statistics
	*N* = 84	*N* = 16			
Count	42.87 ± 37.257^a^	31.80 ± 30.995	0.52	0.323	1.03
Quick & Slow	31.42 ± 18.795	37.00 ± 12.981	0.52	−0.345	−0.71
Motility	40.22 ± 20.067	48.40 ± 11.059	0.37	−0.505	−3.32
Morphology	1.69 ± 1.221	1.40 ± 0.548	0.60	0.307	2.48
Volume	3.380 ± 1.574	3.480 ± 1.028	0.89	−0.075	3.95

^a^ Data are represented as mean ± standard deviation.

## 5 Discussion

The progress and development of men's infertility are influenced by various risk factors and underlying diseases [13, 14, 15]. In many studies, the presence of inflammation in the male reproductive tract, cytokines and receptors indicates the infection [16, 17, 18]. Other factors in infertility are infectious agents such as viruses [19]. In our study, seminal fluid samples from 100 infertile men and 100 fertile men from the Yazd Infertility Research Center were collected and the incidence of CMV as well as HSV infection was evaluated.

Our findings indicate a higher prevalence of HSV infection in infertile men compared to fertile men, with a statistically significant difference (*p* = 0.003). This aligns with previous studies, such as those by El Borai (1998) and Salehi‐vaziri (2010), which observed significant associations between HSV infection and impaired semen parameters [20, 21].

Kurscheidt et al. conducted a study to explore the effects of HSV infection on seminal parameters. To achieve this, they designed a multiplex polymerase chain reaction assay capable of identifying both HSV‐1 and HSV‐2 within semen samples. The investigation involved the analysis of 279 semen samples collected from men undergoing fertility evaluation. The results of the study revealed the presence of HSV in 10.7% of the analyzed samples. Specifically, 7.5% of the samples tested positive for HSV‐1, while 3.2% showed the presence of HSV‐2. Notably, a connection was established between HSV‐2 infection and symptoms like hematospermia and decreased seminal volume. Conversely, HSV‐1 infection was found to be associated with a decline in sperm count. These noteworthy findings indicate that HSV infections in male partners of infertile couples could potentially impact both spermatozoa and various components of seminal fluid, thereby influencing fertility outcomes [22]. Our study was also consistent with this study that the prevalence of HSV infection in infertile men was higher than in fertile ones and the difference between the two groups was statistically significant (*p* = 0.003).

However, it is important to note that some studies have found no association between herpesvirus presence in semen and impaired reproductive outcomes. For instance, Kaspersen and Höllsberg (2013) reported that while herpesviruses are frequently detected in semen, the majority of studies have failed to establish a clear link between HSV presence and reduced sperm quality or fertility. They highlighted that the association between HSV in semen and impaired reproduction remains inconclusive, as many studies do not support this connection. Their review suggests that despite the frequent presence of herpesviruses, there is no firm evidence establishing a direct impact on human reproduction [23]. Our study supports the notion that HSV infection may be a contributing factor to male infertility, as evidenced by our significant findings. Nevertheless, it is crucial to consider the broader context of existing literature, which suggests that the presence of herpesviruses alone might not universally impact semen quality or fertility outcomes. Further research is needed to clarify the potential mechanisms by which herpesviruses might influence male reproductive health and to address the inconsistencies observed in different studies.

In distinct research involving 172 male participants (80 with normal semen and 92 with abnormal semen), the presence of HSV DNA was scrutinized. Remarkably, HSV DNA was identified in 83.1% of the collected semen samples. Among the samples with normal semen parameters, varying rates of detection were observed for HSV‐1, VZV, EBV, CMV, HHV‐6, and HHV‐7. Similarly, HSV was also found in samples exhibiting abnormal semen characteristics. This investigation underscores the frequent occurrence of HSV within semen and underscores the necessity for further exploration into their potential role in male factor infertility [24]. Our study showed that CMV infection had no significant effect on sperm count, progressive sperm, motility, total sperm motility, sperm shape or morphology and seminal fluid volume. Therefore, this study is consistent with other studies, indicating no significant effect of this infection on male fertility. Although it is not unreasonable that this infection could be a risk factor for exacerbating infertility and reducing sperm motility.

Recent studies indicate that severe acute respiratory syndrome coronavirus‐2 (SARS‐CoV‐2) can affect organs beyond the respiratory system, including the digestive and reproductive systems. The virus can enter the male reproductive system through receptors on the testes, impacting various aspects of it [25]. Specifically, SARS‐CoV‐2 enters target cells via the ACE2‐TMPRSS2 receptor, which is more prevalent on the surface of the male reproductive system compared to females, potentially explaining the higher male susceptibility, with a male‐to‐female ratio of 2.7:1 [26, 27]. Research has increasingly focused on the implications of SARS‐CoV‐2 on men's reproductive health and fertility. A study by Li H. on sperm samples from 23 COVID‐19 patients found a decrease in sperm concentration compared to the control group, along with observed spermatogenesis disorders [28]. Similarly, a systematic review by Y. He et al. (2023) reported a decline in sperm quality, including count, in COVID‐19 patients [29]. Holtmann's study further compared semen parameters in COVID‐19 patients with a control group, revealing a reduction in sperm motility and count in patients with moderate symptoms, though these changes were not observed in those with mild symptoms [30]. A systematic review by Tufvesson et al. (2022) highlighted the significant impact of SARS‐CoV‐2 on semen quality, particularly within the first 3 months post‐diagnosis. The review, which included nine cohort studies, found a statistically significant decrease in all semen parameters when samples were collected less than 3 months after diagnosis. However, no significant differences were observed when semen was analyzed more than 3 months after recovery [27]. These findings underscore the importance of considering the timing of infection when assessing the virus's impact on semen quality, and they suggest that the effects of SARS‐CoV‐2 on the male reproductive system warrant further investigation.

## 6 Conclusion

Our focus was centered on the prevalence and impact of infectious agents within the reproductive system, with particular emphasis on CMV and HSV, comparing their occurrence between fertile and infertile men. The investigation also encompassed an evaluation of sperm quality indices, coupled with an exploration of the influence of various factors in relation to viral infections. Regarding CMV prevalence, our results revealed no significant disparity between the fertile and infertile groups, indicating the absence of a pronounced viral outbreak within the infertile patient population. Furthermore, our analysis unveiled no substantial correlation between CMV infection and sperm quality variables, suggesting that this viral agent may not significantly affect sperm quality in the studied context.

In our study, HSV infection demonstrated a more prevalent presence among infertile individuals, highlighting a significant association. This infection notably influenced both sperm count and morphology, showcasing its impactful role in these aspects while displaying no noteworthy effect on other variables under scrutiny. This study shows HSV infection as a potential risk factor in male infertility development and progression, reaffirming earlier findings. It is essential to acknowledge that while CMV infection did not exhibit a pronounced impact in this study, the potential exacerbating role of this infection on the issue of male infertility should not be entirely discounted.

## Author Contributions

**Seyed Mansour Salar:** methodology, writing–original draft, investigation, validation. **Fahime Edalat:** methodology, investigation, writing–original draft, validation. **Arash Letafati:** writing–original draft, investigation, methodology, validation. **Seyed Mehdi Kalantar:** investigation, validation, writing–review and editing. **Fatemeh Javanmardi:** software, formal analysis, data curation. **Ahmad Najafi:** writing–review and editing, investigation, validation. **Mahmoud vakili:** investigation, writing–review and editing, validation. **Afagh Moattari:** conceptualization, supervision, funding acquisition, validation, project administration.

## Disclosure

All authors have read and approved the final version of the manuscript. The corresponding author had full access to all of the data in this study and takes complete responsibility for the integrity of the data and the accuracy of the data analysis. The lead author Afagh Moattari affirms that this manuscript is an honest, accurate, and transparent account of the study being reported; that no important aspects of the study have been omitted; and that any discrepancies from the study as planned (and, if relevant, registered) have been explained.

## Ethics Statement

Ethical approval has been obtained from Sahahid Sadoughi University of Medical Sciences (IR.SSU.RSI.REC.1395.32). (IR. SSU.RSI.REC.1395.32). Also, informed consent was obtained from all subjects or their legal guardians.

## Conflicts of Interest

The authors declare no conflict of interest. The funding source had no role in the study design; collection, analysis, and interpretation of data; writing of the report; or the decision to submit the report for publication.

## Data Availability

The datasets used and/or analyzed during the current study available from the corresponding author on reasonable request.
